# Supplementary material for: Natural Infections of Potato Plants Grown from Minitubers with Blackleg-Causing Soft Rot Pectobacteriaceae
Source: Microorganisms. 2022 Dec 17;10(12):2504. doi: 10.3390/microorganisms10122504 (PMC9787864; doi:10.3390/microorganisms10122504)
Supplement: Supplementary file 1 [file microorganisms-10-02504-s001.zip › FigureS1 FAM versus other labels.pptx]

## Slide 1
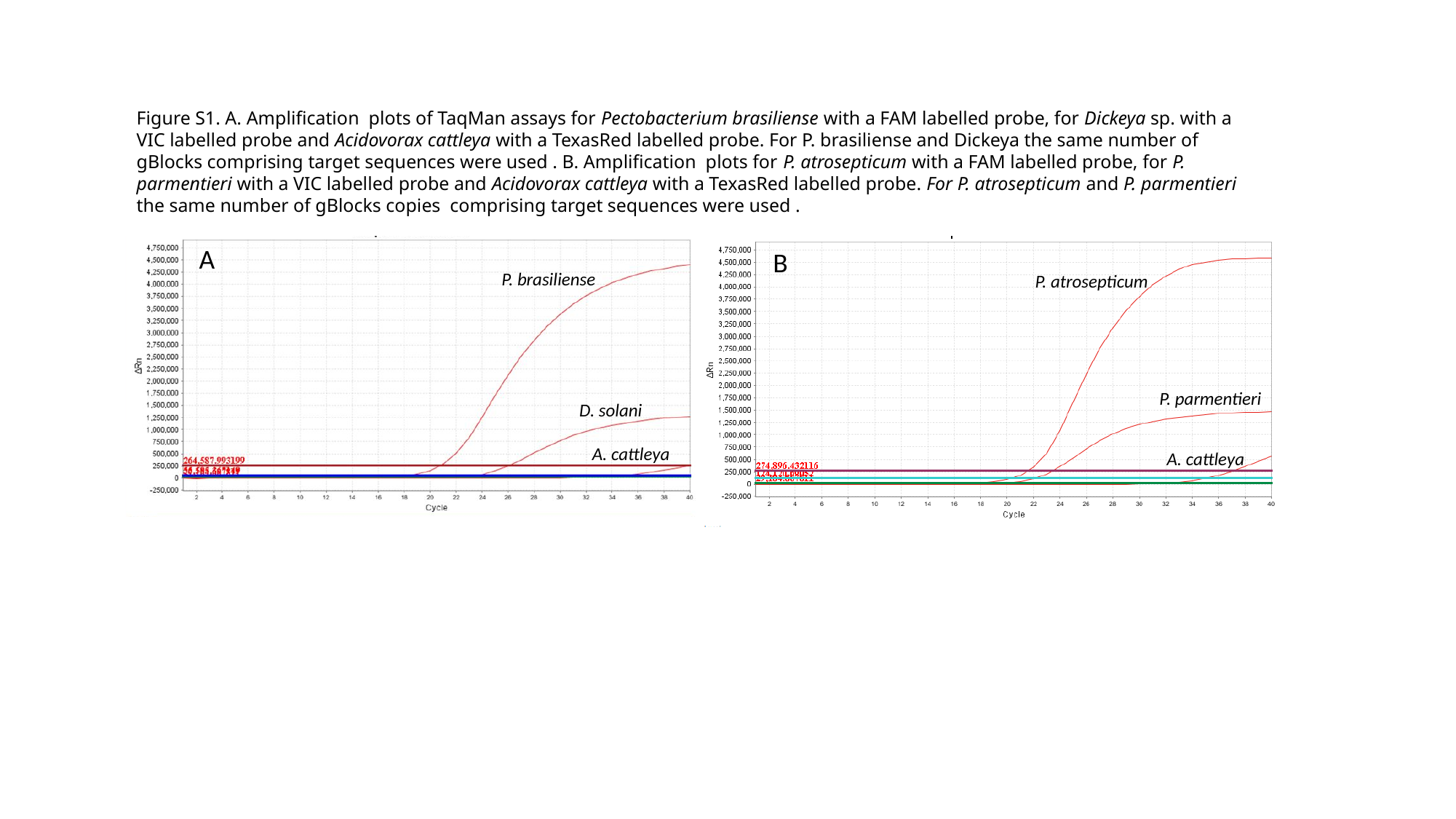

Figure S1. A. Amplification plots of TaqMan assays for Pectobacterium brasiliense with a FAM labelled probe, for Dickeya sp. with a VIC labelled probe and Acidovorax cattleya with a TexasRed labelled probe. For P. brasiliense and Dickeya the same number of gBlocks comprising target sequences were used . B. Amplification plots for P. atrosepticum with a FAM labelled probe, for P. parmentieri with a VIC labelled probe and Acidovorax cattleya with a TexasRed labelled probe. For P. atrosepticum and P. parmentieri the same number of gBlocks copies comprising target sequences were used .
A
B
P. brasiliense
P. atrosepticum
P. parmentieri
D. solani
A. cattleya
A. cattleya
