# Supplementary material for: Natural Infections of Potato Plants Grown from Minitubers with Blackleg-Causing Soft Rot Pectobacteriaceae
Source: Microorganisms. 2022 Dec 17;10(12):2504. doi: 10.3390/microorganisms10122504 (PMC9787864; doi:10.3390/microorganisms10122504)
Supplement: Supplementary file 1 [file microorganisms-10-02504-s001.zip › FigureS2multiplexTaqMan_rev.pptx]

## Slide 1
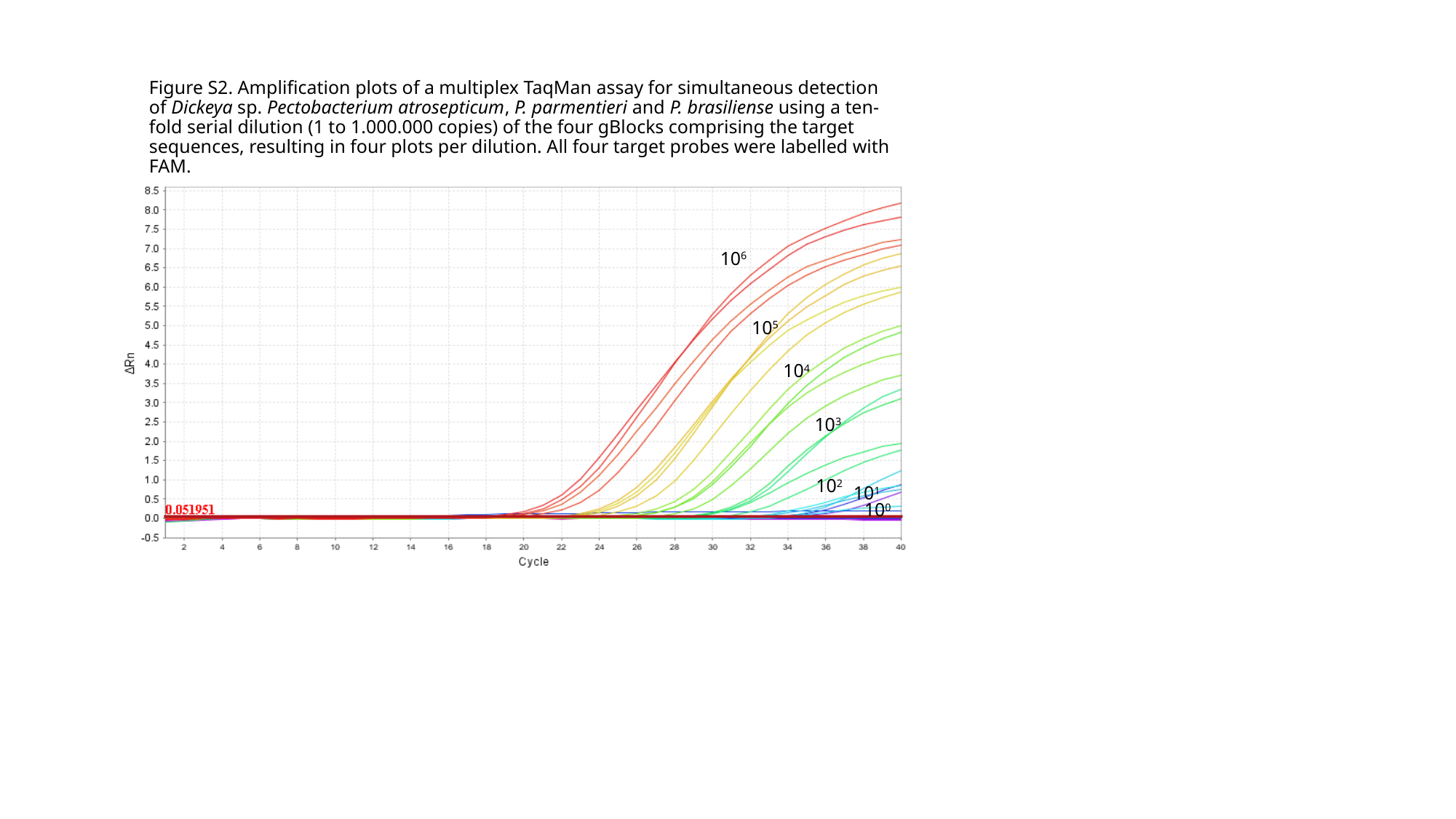

# Figure S2. Amplification plots of a multiplex TaqMan assay for simultaneous detection of Dickeya sp. Pectobacterium atrosepticum, P. parmentieri and P. brasiliense using a ten-fold serial dilution (1 to 1.000.000 copies) of the four gBlocks comprising the target sequences, resulting in four plots per dilution. All four target probes were labelled with FAM.
106
105
104
103
102
101
100
