# Supplementary material for: Natural Infections of Potato Plants Grown from Minitubers with Blackleg-Causing Soft Rot Pectobacteriaceae
Source: Microorganisms. 2022 Dec 17;10(12):2504. doi: 10.3390/microorganisms10122504 (PMC9787864; doi:10.3390/microorganisms10122504)
Supplement: Supplementary file 1 [file microorganisms-10-02504-s001.zip › FigureS3 Ct-values2019.pptx]

## Slide 1
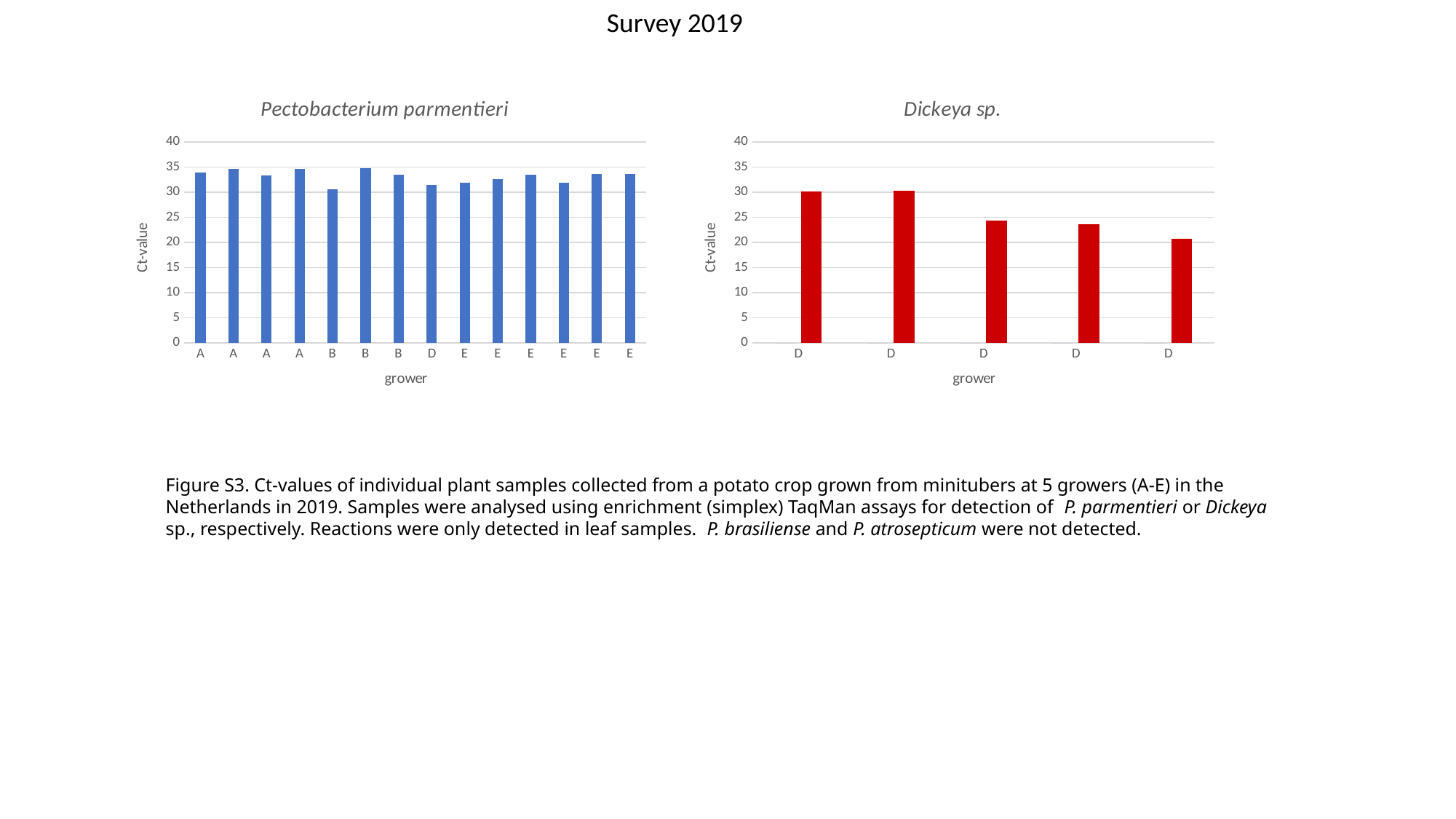

Survey 2019
### Chart: Pectobacterium parmentieri
| Category | |
|---|---|
| A | 33.98 |
| A | 34.67 |
| A | 33.39 |
| A | 34.64 |
| B | 30.57 |
| B | 34.8 |
| B | 33.42 |
| D | 31.39 |
| E | 31.83 |
| E | 32.54 |
| E | 33.44 |
| E | 31.92 |
| E | 33.57 |
| E | 33.57 |
### Chart: Dickeya sp.
| Category | | |
|---|---|---|
| D | 0.0 | 30.11 |
| D | 0.0 | 30.3 |
| D | 0.0 | 24.34 |
| D | 0.0 | 23.66 |
| D | 0.0 | 20.74 |Figure S3. Ct-values of individual plant samples collected from a potato crop grown from minitubers at 5 growers (A-E) in the Netherlands in 2019. Samples were analysed using enrichment (simplex) TaqMan assays for detection of P. parmentieri or Dickeya sp., respectively. Reactions were only detected in leaf samples. P. brasiliense and P. atrosepticum were not detected.
