# Supplementary material for: Natural Infections of Potato Plants Grown from Minitubers with Blackleg-Causing Soft Rot Pectobacteriaceae
Source: Microorganisms. 2022 Dec 17;10(12):2504. doi: 10.3390/microorganisms10122504 (PMC9787864; doi:10.3390/microorganisms10122504)
Supplement: Supplementary file 1 [file microorganisms-10-02504-s001.zip › TableS3 Ctvaluesvs2.docx]

Table S3. Median and range of Ct-values of samples positive (Ct < 35) in enrichment TaqMan assays^1^ for *Dickeya* species, *Pectobacterium brasiliense* or *P. parmentieri* in potato tuber, stem or leaf extracts. Samples were collected from a crop at the end of the growing season of 2019 and 2020 grown from mini-tubers.

|  | **Tubers** | | **Stems** | | **Leaves** | |
| --- | --- | --- | --- | --- | --- | --- |
|  | **Median** | **Range** | **Median** | **Range** | **Median** | **Range** |
| ***Survey 2019*** |  |  |  |  |  |  |
| *Dickeya* spp. | NP^2^ | NP | NP | NP | 24.3 | 20.8 – 30.3 |
| *P. brasiliense* | NP | NP | NP | NP | NP | NP |
| *P. parmentieri* | NP | NP | 33.4 | 30.6 – 34.8 | 33.5 | 33.4 – 33.7 |
|  |  |  |  |  |  |  |
| ***Survey 2020*** |  |  |  |  |  |  |
| *Dickeya* spp. | 23.8 | 17.2 - 34.4 | 33.8^3^ | ND | ND | ND |
| *P. brasiliense* | 27.4 | 19.7 – 34.9 | 22.5 | 16.5 – 31.1 | 25.5 | 15.3 – 33.2 |
| *P. parmentieri* | 29.2 | 23.1 - 34.9 | ND^4^ | ND | 32.9 | 32.3 - 33.3 |

^1^ Samples were enriched for soft rot Pectobacteriaceae in a medium under anaerobic conditions that allowed selective multiplication of the target bacteria.

^2^ NP = no positive samples present

^3^ Only 1 sample positive

^4^ ND = not determined
