# Supplementary figures and images for: Natural Infections of Potato Plants Grown from Minitubers with Blackleg-Causing Soft Rot Pectobacteriaceae
Source: Microorganisms. 2022 Dec 17;10(12):2504. doi: 10.3390/microorganisms10122504 (PMC9787864; doi:10.3390/microorganisms10122504)

## Slide 1
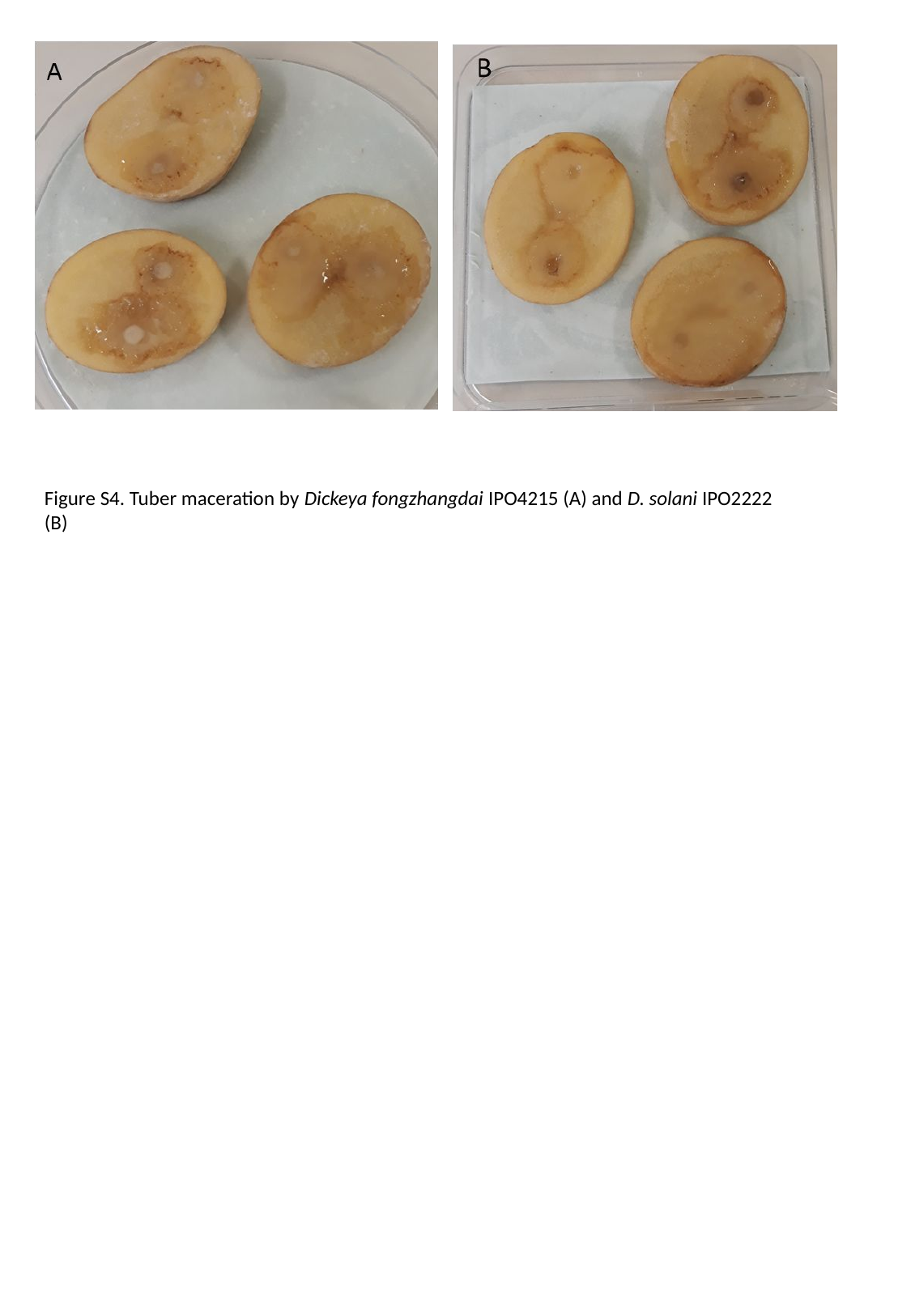

Figure S4. Tuber maceration by Dickeya fongzhangdai IPO4215 (A) and D. solani IPO2222 (B)

Supplement: Supplementary file 1 [file microorganisms-10-02504-s001.zip › FigureS4 Tubermaceration.pptx]
